# CAP Analysis of the Distribution of the Introduced *Bemisia tabaci* (Hemiptera: Aleyrodidae) Species Complex in Xinjiang, China and the Southerly Expansion of the Mediterranean Species

**DOI:** 10.1093/jisesa/ieaa151

**Published:** 2021-04-12

**Authors:** Zunzun Jia, Kaiyun Fu, Wenchao Guo, Weihua Jiang, Tursun Ahmat, Xinhua Ding, Jiang He, Xiaowu Wang

**Affiliations:** 1 College of Agriculture, Xinjiang Agricultural University, Xinjiang, China; 2 Institute of Plant Protection, Xinjiang Academy of Agricultural Sciences/Key Laboratory of Integrated Pest Management on Crops in Northwestern Oasis, Ministry of Agriculture/Scientific Observing and Experimental Station of Korla, Ministry of Agriculture, Xinjiang, China; 3 Institute of Microbial Application, Xinjiang Academy of Agricultural Sciences, Xinjiang, China; 4 College of Plant pretection, Nanjing Agricultural University, Nanjing, China

**Keywords:** *Bemisia tabaci*, cryptic species, invasive species, genetic diversity

## Abstract

*Bemisia tabaci* (Gennadius) cryptic complex has invaded Xinjiang, China, since 1998. The distribution of Mediterranean (MED) and Middle East-Asia Minor 1 (MEAM1) *B. tabaci* substrains has been gradually identified due to the development of molecular technology. In this study, the distribution of MED and MEAM1 in Xinjiang was determined by cleaved amplified polymorphic sequence (CAPs). Results showed that MED dominated in northern Xinjiang (84%), whereas MEAM1 was dominant in southern Xinjiang (72%). Five pairs of simple sequence repeat (SSR) primers were used to analyze the genetic diversity of *B. tabaci* among 36 geographic populations. The genetic diversity of MED and MEAM1was low and varied little among populations in Xinjiang (0.09 ± 0.14 and 0.09 ± 0.13, respectively). Based on ∆*K* statistic, 13 populations of MEAM1 could be classified into two subgroups at *K* = 2, whereas the 23 populations of MED could be classified into four subgroups at *K* = 4. However, Mantel *t*-test demonstrated no correlation between geographical and genetic distances among *B. tabaci* complex (*R* = 0.42, *P* = 1.00). Neighbor-joining and principal coordinate analysis showed that geographical isolation and interspecific differences were the main causes of the genetic variation. Gene flow predicted that MEAM1 was most likely introduced from Urumqi to the southern Xinjiang. Meanwhile, a large proportion of MED in Kashi region came from Changji and Yining. To block ongoing dispersal, strict detection and flower quarantine regulations need to be enforced.

The whitefly, *Bemisia tabaci* (Gennadius), is a rapidly evolving cryptic species complex (Perring 2001), composed of at least 34 cryptic species (Boykin and De Barro 2012, Alemandri et al. 2015, Götz and Winter 2016). Among the complex members, Middle East-Asia Minor 1 (MEAM1, previously known as B biotype) and Mediterranean (MED, previously known as Q biotype) are the most adaptive whitefly cryptic species in the world. Both cause severe damage to feeding crops and horticultural plants, e.g., nectar secretion leading to sooty mold, and transmission of various viruses (Judith and Czosnek 2002, Paul and Barro 2012, Navas-Castillo 2014, Gilbertson et al. 2015, Wainaina et al. 2018), including cotton leaf curl Multan virus (CLCuMuV), which causes a significant reduction in yield (Nur and Abu Salih 1970, Briddon and Markham 2000, Tahir et al. 2011, Chatzivassiliou et al. 2007).

As an important invasive pest, *B. tabaci* first invaded China in 1949 (Zhou 1949). In the 1990s, *B. tabaci* brought serious damage to various regions (Zhang 2000, Chu et al. 2010, Wang et al. 2010, Guo et al. 2014). In 1998, *B. tabaci* first settled in the flower market in Urumqi, and it soon spread to Shihezi, Hami, Korla, Karamay, and Turpan (Zhao et al. 2000). Before 2010, MEAM1 was the sole species identified in Xinjiang (Zhao et al. 2000). Using sequence characterized amplified regions (SCAR) and *COI* (cytochrome oxidase I) barcoding, Cao et al. (2011) found out that MED had invaded Xinjiang.

Recently, molecular markers such as *COI* barcoding and simple sequence repeat (SSR) have been widely used to identify species of whiteflies. These tools help in understanding genetic diversity, gene flow, and even the replacement of populations (David 1998, Götz and Winter 2016). Boykin et al. (2007) used the whole *COI* sequence to analyze the genetic diversity of the *B. tabaci*, and he unveiled 12 members of the species complex. With the discovery of MED *B. tabaci*’s invasion, it was then known that Xinjiang harbored at least two cryptic species: MED and MEAM1 (Duan et al. 2011).

SSR is widely used in identification and in investigations related to the genetic evolution, genetic mapping, phylogenetic, and genetic diversity of animal and plant species. For example, Colorado potato beetles, *Leptinotarsa decemlineata* (Say) (Coleoptera: chrysomelidae), was a notoriously invasive species. Zhang et al. (2013) analyzed its genetic diversity among 10 geographical populations in Xinjiang and the mode of the beetle diffusion from the border to inner Xinjiang. Previous report on *B. tabaci* showed that SSR may not distinguish cryptic species in the complex unless there was an appropriate method to study population structure and the mechanism of invasion and diffusion (Chu et al. 2012). Thus, cleaved amplified polymorphic sequence (CAPs) was used to detect cryptic species within the *B. tabaci* species complex. Previous COI barcoding studies showed that only MEAM1 and MED of *B. tabaci* were found in Xingjiang (Ashfaq et al. 2015).

With the expansion of greenhouses and the increasing frequency of flower trade, whiteflies have invaded most areas in Xinjiang (Jia et al. 2017). As the largest cotton-producing area in China, Xinjiang is facing great threats of the whitefly-transmitted cotton leaf curl Multan virus. Despite of this, the distribution of MED and MEAM1 *B. tabaci* remains unclear. Meanwhile, the ecological conditions of the unique desert oasis agriculture in Xinjiang may impose certain limitations on the spread of whitefly. In this study, we aimed to uncover the distribution patterns of MEAM1 and MED from 2015 to 2017 to provide insights into the invasion and diffusion of the *B. tabaci* complex in Xinjiang.

## Materials and Methods

### Samples

*Bemisia tabaci* samples were collected from 54 sites in Xinjiang from June 2015 to December 2017 (Table 1). At least 200 adults per site were collected, placed in tubes containing 95% ethanol, and stored at −20°C. Thirty to 50 samples were randomly selected for identification. In total, 1,768 adults were identified as MEAM1 or MED cryptic species. We chose samples for SSR by initially, randomly sampling the sites, and then by the individuals coming from the chosen sites (Table 1).

**Table 1.** Sampling information for *Bemisia tabaci*collected in Xinjiang

Area	No.	Population	Time	Longitude and latitude	Host	No.	No. of SSR
Urumqi	1	Urumqi-1	2016.5	(43°48′49″N, 87°34′37″W)	*Pharbitis nil*	28	0
	2	Urumqi-2	2016.8	(43°48′21″N, 87°33′22″W)	*Cucumissativus*	50	49
					Subtotal	78	49
Karamay	3	Karamay	2016.5	(45°29′26″N, 84°57′23″W)	*Solanum lycopersicum*	50	40
Hami City	4	Hami-1	2016.4	(42°50′32″N, 93°27′18″W)	*Solanum lycopersicum*	30	16
	5	Hami-2	2016.4	(43°0′35″N, 93°35′58″W)	*Solanum lycopersicum*	30	24
					Subtotal	60	40
Changji hui autonomous prefecture	6	Changji-1	2016.5	(44°0′40″N, 87°18′29″W)	*Salvias plendens*	60	41
	7	Changji-2	2017.3	(44°9′32″N, 88°1′22″W)	*Hibiscus rosa-sinensis*	15	0
	8	Changji-3	2017.3	(44°9′32″N, 87°58′45″W)	*Hibiscus rosa-sinensis*	15	0
					Subtotal	90	41
Bortala Mongol Autonomous Prefecture	9	Alashankou	2016.9	(45°10′12″N, 82°33′36″W)	*Hibiscus rosa-sinensis*	50	50
Mongolian Autonomous	10	Bazhou-1	2015.8	(38°7′4″N, 85°34′46″W)	*Vignaunguiculata*	30	28
Prefecture of Bayingolin	11	Bazhou-2	2015.8	(38°8′3″N, 85°30′42″W)	*Vignaunguiculata*	30	0
	12	Bazhou-3	2015.8	(38°6′10″N, 85°31′9″W)	*Cucurbita moschata*	30	19
	13	Korla	2016.3	(41°46′12″N, 86°8′57″W)	*Hibiscus rosa-sinensis*	30	0
					Subtotal	120	47
AksuPrefecture	14	Aksu	2017.3	(40°32′19″N,81°17′53″W)	*Hibiscus rosa-sinensis*	30	0
Kashgar Prefecture	15	Kashi-1	2015.9	(38°24′44″N, 77°17′3″W)	*Gossypiumspp*	50	33
	16	Kashi-2	2015.7	(39°24′0″N, 75°54′0″W)	*Solanum lycopersicum*	30	11
	17	Kashi-3	2015.8	(38°21′22″N, 77°10′56″W)	*Gossypiumspp*	30	11
	18	Kashi-4	2015.8	(38°17′9″N, 77°11′49″W)	*Gossypiumspp*	30	6
	19	Kashi-5	2015.8	(38°19′20″N, 77°12′10″W)	*Gossypiumspp*	30	9
	20	Kashi-6	2015.8	(38°20′44″N, 77°09′26″W)	*Gossypiumspp*	30	4
	21	Kashi-7	2015.9	(39°24′35″N, 76°8′1″W)	*solanum melongena*	30	5
	22	Kashi-8	2015.9	(39°24′21″N, 76°5′3″W)	*solanum melongena*	30	10
	23	Kashi-9	2015.9	(39°14′3″N, 76°22′35″W)	*Vignaunguiculata*	30	0
	24	Kashi-10	2015.9	(38°55′41″N, 76°8′13″W)	*Cucumissativus*	30	0
	25	Kashi-11	2015.9	(39°47′0″N, 78°31′46″W)	*solanum melongena*	30	0
	26	Kashi-12	2015.9	(39°47′41″N, 78°34′18″W)	*Cucumissativus*	30	0
	27	Kashi-13	2016.4	(38°18′28″N, 77°56′42″W)	*Solanum lycopersicum*	30	0
					Subtotal	410	89
Hotan Prefecture	28	Hetian-1	2015.6	(37°27′47″N, 79°52′29″W)	*Solanum lycopersicum*	60	0
	29	Hetian-2	2015.8	(37°4′4″N, 82°42′55″W)	*Vignaunguiculata*	30	10
	30	Hetian-3	2015.8	(37°4′6″N, 82°40′11″W)	*Canavaliagladiata*	30	0
	31	Hetian-4	2015.8	(37°3′29″N, 82°41′41″W)	*Solanum lycopersicum*	30	6
	32	Hetian-5	2015.8	(36°50′16″N, 81°38′51″W)	*Solanum lycopersicum*	30	14
	33	Hetian-6	2014.8	(36°49′1″N, 81°38′23″W)	*Solanum lycopersicum*	30	5
	34	Hetian-7	2015.8	(36°50′58″N, 81°37′8″W)	*solanum melongena*	30	0
	35	Hetian-8	2015.8	(36°59′55″N, 81°4′12″W)	*Capsicum annuum*	30	6
	36	Hetian-9	2015.8	(36°59′49″N, 80°58′51″W)	*Brassica rapa*	30	12
	37	Hetian-10	2015.8	(37°14′14″N, 79°41′34″W)	*Brassica rapa*	30	0
	38	Hetian-11	2015.8	(37°14′18″N, 79°44′19″W)	*Solanum lycopersicum*	30	5
	39	Hetian-12	2015.8	(37°17′52″N, 79°39′45″W)	*Cucurbita moschata*	30	5
	40	Hetian-13	2016.4	(37°15′7″N, 80°5′12″W)	*Solanum lycopersicum*	60	27
					Subtotal	450	90
Ili Kazak Autonomous Prefecture	41	Yining-2015	2015.5	(43°51′28″N, 81°25′56″W)	*Capsicum annuum*	30	0
	42	Yining-2016	2016.3	(43°51′29″N, 81°25′56″W)	*Capsicum annuum*	50	50
					Subtotal	80	50
Turpan	43	Tulupan-1	2015.6	(42°59′59″N, 89°9′58″W)	*Solanum lycopersicum*	30	0
	44	Tulupan-2	2015.6	(42°57′56″N, 89°6′16″W)	*Cucumber*	30	10
	45	Tulupan-3	2015.6	(42°48′40″N, 88°27′17″W)	*Gossypiumspp*	30	
	46	Tulupan-4	2016.4	(42°46′54″N, 88°41′56″W)	*Solanum lycopersicum*	30	13
	47	Tulupan-5	2016.4	(42°79′37″N, 88°38′24″W)	*Solanum lycopersicum*	30	18
	48	Tulupan-6	2016.4	(42°57′6″N, 89°5′22″W)	*Solanum lycopersicum*	30	14
	49	Tulupan-7	2016.4	(42°46′39″N, 89°33′31″W)	*Solanum lycopersicum*	30	18
	50	Tulupan-8	2016.4.	(42°47′14″N, 89°43′18″W)	*Solanum lycopersicum*	30	11
	51	Tulupan-9	2016.4	(42°43′21″N, 89°41′43″W)	*Pharbitis nil*	30	8
					Subtotal	270	92
Shihez	53	Shihezi	2016.5	(44°18′22″N, 86°4′48″W)	*Euphorbia pulcherrima*	50	43
Alaer	54	Alaer	2017.3	(40°32′42″N, 81°16′41″W)	*Hibiscus rosa-sinensis*	30	0
					Total	1,768	631

### Cleaved Amplified Polymorphic Sequence

Total genomic DNA was extracted from each adult according to Frohlich et al. (2010). Briefly, each adult was laid on clean parafilm and homogenized in DNase-free Eppendorf tube (Thermo Fisher Scientific) containing 5-µl lysis buffer (5 mM Tris [pH 8.0], 0.5 mM EDTA, 0.5% NP40, and 1 mg/ml protein K^+^; Beijing Solarbio Science & Technology Co., Ltd.). The homogenate was then transferred to tubes with 30-µl lysis buffer and incubated at 65°C for 15 min and then at 95°C for 10 min. The products were stored at −20°C.

A 13-µl reaction system containing 2.6-µl DNA, 6.5-µl 2× Taq PCR Master Mix (Tiangen Biotech Co., Ltd. Beijing), and 0.52-µl primer (20 µM, Nanjing Jinsirui Biotechnology Co., Ltd.) was predenaturated at 95°C for 5 min, followed by 40 cycles of denaturation at 95°C for 30 s. After annealing at 52°C for 30 s and extension at 72°C for 30 s, samples went through a final extension at 72°C for 5 min. All PCR products were stored at −20°C. The forward and reverse primers were C1-J-2195 (5′-TTGATTTTTTGGTCATCCAGAAGT-3′) and R-BQ-2819 (5′-CTGA-ATATCGRCGAGGCATTCC-3′), respectively (Simon et al. 1994, Chu 2010).

Enzyme digestion method followed Horowitz et al. (2005) and Chu (2010). Seven microliters of VspI Digestion Mix (0.5 µl VspI, 4.5 µl H_2_O, 2 µl buffer 0; Thermo Fisher Scientific) was added to each of the 20-µl PCR products, and the final mixture was incubated at 37°C for 2 h. Digestion products were electrophoresed on 1% agarose gel under 120 V for 45 min and visually inspected under BIO-RAD Gel Doc XR+. MEAM1 individuals were identified by the presence of a unique 620-bp band, whereas MED individuals exhibited 498- and 122-bp bands.

### Simple Sequence Repeat

Five microsatellite markers were selected according to previous studies of De Barro et al. (2003) and Valle et al. (2012; Table 2). The 25-µl PCR system contained 2-µl DNA, 12.5 µl 2× Taq PCR MaserMix (Tiangen Biotech [Beijing] Co., Ltd.), and 1 µl of each primer (20 µM). The PCR program was set to the following conditions: initiation at 95°C for 5 min, followed by 45 cycles of denaturation at 95°C for 30 s, annealing for 30 s, extension at 72°C for 30 s, then final extension at 72°C for 5 min. Annealing temperatures for each primer are provided in Table 2. The PCR products were analyzed by capillary electrophoresis following the directions of DNF-905 kiton Fragment Analyzer TM (Advanced Analytic Service). Fragment detailed data were extracted using Agilent PROSize 2.0.

**Table 2.** The information of simple sequence repeat molecular marker primer

Microsatellite locus	Direction	Primer sequences	Annealing temperature (°C)	References
bem31	F	GTCATTTCTGGATTCTCAGCA	57	De Barro et al. (2003)
	R	AAGAACTAGCCAGGGACAAAC		
bem40	F	GAAAGTGGAGAGTTTAGGTGA	57	De Barro et al. (2003)
	R	TGGAGAATGTTATAAAGTGGA		
BEM06	F	GATGGCTTATGTTATAATACTA	52	De Barro et al. (2003)
	R	TTACACTTAACACCAGAACT		
bta4	F	CGGCAGTCAGGGTTATT	56	Valle et al. (2012)
	R	CGCTCCTCAAGTTTTCTGT		
bta5	F	GCGTAGGAGAGTTGGAATGC	60	Valle et al. (2012)
	R	TATACTTGGGCATCGTCAGC		

### Genetic Diversity

PopGene32 was used to analyze genetic diversity parameters such as the number of alleles (*N*_a_), number of effective alleles (*N*_e_), average diversity index (*H*), and the Shannon diversity index (*I*). The inbreeding coefficients (*F*_IS_) were also calculated to reflect the inbreeding status of the populations. Genetic differences among populations were evaluated by pairwise *F*_IS_ values, with 10 000 permutations to assess significance (Weir and Cockerham 1984; FSTAT v.2.9.3.2, Goudet 2002).

### Population Relationships

Group and population clusters were generated using STRUCTURE v.2.3.3 based on Bayesian methods (STRUCTURE v.2.3.3, Pritchard et al. 2000). Δ*K* method was used to estimate the optimum number of genetic groups (Evanno et al. 2005). We chose the admixture model with correlated allele frequencies and set the *K* from 1 to 10 for MED group and from 1 to 20 for MEAM1 group. We ran three replicates of each run and set a burn-in period of 50 000 Markov chain Monte Carlo generations followed by 5 × 10^5^ iterations. To confirm the existence of population structure, analysis of molecular variance (AMOVA) was performed to assess the genetic variance partitioned into four levels (among groups, among populations, within groups, within populations, and within individuals) based on structure output, with 10 000 permutations to test for significance (Arlequin v.3.11, Excoffier et al. 2005). Correlation between geographical and genetic distances (Slatkin’s linear *F*_ST_, *F*_ST_/(1 − *F*_ST_), and *D*′) of all samples from Xinjiang as well as between and within groups were defined by Structure (Mantel procedure, GenAlEx 6.2; Peakall and Smouse 2006). We used a neighbor-joining cluster method in MEGA 3.01 to generate a circle phylogenetic tree based on *D*′ distance which was calculated using Popgene32. To assess the genetic associations of individuals among various groups representing cryptic species and population, a pairwise similarity matrix was generated using simple matching coefficient (Sokal and Michener 1958), with 10 000 permutations. Random, this was converted to Euclidean distance matrix as the square root of 1 minus element-wise similarity for a principal coordinate analysis (PCoA) using NTSYS-pc 2.01 (Rohlf 1987). Top 2 components were plotted for MED, MEAM1, MED, and MEAM1 complex. Direction of gene flow was inferred by a partial Bayesian method from GENECLASS 2.0, and assignment probabilities of individuals were tested from geographical populations with a threshold probability value of 0.01. Assigned probability data of MED to MED, MED to MEAM1, MEAM1 to MED, MEAM1 to MEAM1 groups were arcsin-square root transformed to normal distribution. Significant levels (*P* < 0.05) were tested using Tukey’s multiple comparisons test of ANOVA in Graphpad Prism 6.0 (Rannala and Mountain 1997) and GeneClass v.2.0 (Paetkau et al. 2004, Piry et al. 2004).

## Results

### The Distribution of *B. tabaci* Cryptic Species

**Fig. 1.**
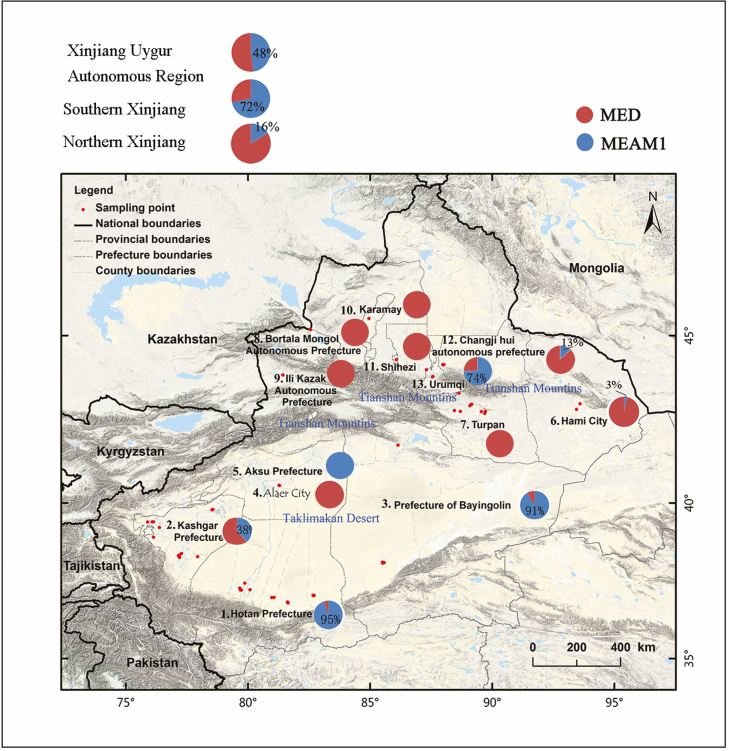
Sampling for *Bemisia tabaci* collected in Xinjiang.

**Fig. 2.**
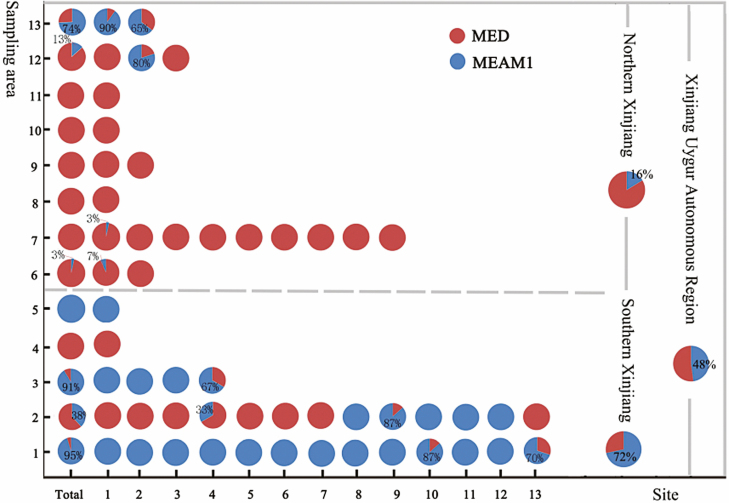
Distribution of 54 populations of *Bemisia tabaci* cryptic species in Xinjiang. Sampling sites: 1. Hetian Prefecture; 2. Kashgar Prefecture; 3. Mongolian Autonomous Prefecture of Bayingolin; 4. Alaer; 5. Aksu Prefecture; 6. Hami City; 7. Turpan; 8. Bortala Mongol Autonomous Prefecture; 9. Ili Kazak Autonomous Prefecture; 10. Karamay; 11. Shihez; 12. Changji hui autonomous prefecture; 13. Urumqi.

The distribution of *B. tabaci* cryptic species were identified in 54 sampling sites during 2015 and 2017 by CAPs. The randomly chosen 631 samples for SSR were also sequenced to verify these results (data not shown). Results showed that there was a large percentage difference between northern and southern Xinjiang (Fig. 1). The MED was the dominant cryptic species in northern Xinjiang, accounting for 84% of the samples. Apart for Urumqi (26%), it dominated in Changji Hui Autonomous Prefecture (87%), Shihezi City (100%), Karamay City (100%), Yili District (100%), Bortala Mongolian Autonomous Prefecture (100%), Turpan Region (100%), and Hami Region (97%). Meanwhile, the MEAM1 was the dominant cryptic species in southern Xinjiang, accounting for 73% of the samples. It dominated in Hotan (95%), Bazhou (91%), Kashgar (38%), and Aksu (100%). No species were present in Alar (0%; Fig. 2).

### Genetic Diversity

Five pairs of SSR primers were used to analyze the genetic diversity of 631 samples belonging to 36 randomly selected *B. tabaci* populations (Table 1). Polymorphism position and polymorphism information content, ranging from 15 to 48 and 0.92 to 0.95, respectively, showed high loci polymorphism in each population. The results also showed that the primers used were applicable for genetic diversity analysis of *B. tabaci*. The MEAM1 and MED population’s diversity indices including number of alleles per locus (*n*_a_), effective number of alleles (*n*_e_), gene diversity (*h*), and Shannon’s Information index (*I*) averaged at 1.77 ± 0.42 and 1.92 ± 0.27 (range 1.15–1.48), 1.13 ± 0.25 and 1.13 ± 0.25 (range 1.07–1.13), 0.09 ± 0.14 and 0.09 ± 0.13 (range from 0.04 to 0.08), and 0.15 ± 0.20 and 0.15 ± 0.20 (range 0.07–0.13), respectively. These indicates low genetic diversity and little variation among populations (Table 2). *F*_is_ values were all negative in 36 SSR analyzed sites with 12 and 20 populations reaching *P* < 0.05 and *P* < 0.001 significant departure levels. These results indicate that the genetic diversity of *B. tabaci* in Xinjiang is low and the genetic differentiation is high.

### Genetic Relationship

#### ∆*K* Statistic

Based on ∆*K* statistic, 13 populations of MEAM1 could be divided into two subgroups at *K* = 2, whereas the 23 populations of MED could be divided into four subgroups at *K* = 4 (Table 3). In MEAM1, one group Kashi-1, Kashi-2, Kashi-3 included those from Tulupan; the third group included those from Changji and Yining; and finally, the fourth group included those from Alashankou, Shihezi, and Hami.

**Table 3.** Proportion of membership of each predefined population in MEAM1 or MED groups

Population	*K* = 2, *P* = 13		Population	*K* = 4, *P* = 23			
MEAM1	1	2	MED	1	2	3	4
Urumqi-2-MEAM1	0.25	**0.75**	Kelamayi-MED	0.05	0.02	**0.88**	0.05
Hetian-13-MEAM1	0.04	**0.96**	Urumqi-2-MED	0.32	0.02	**0.57**	0.09
Hetian-2-MEAM1	0.05	**0.96**	Kashi-2-MED	0.03	0.08	**0.85**	0.04
Hetian-4-MEAM1	0.04	**0.96**	Kashi-1-MED	0.03	0.03	**0.90**	0.05
Hetian-5-MEAM1	0.03	**0.97**	Kashi-3-MED	0.03	0.02	**0.93**	0.01
Hetian-6-MEAM1	0.04	**0.96**	Kashi-4-MED	0.02	0.03	**0.94**	0.02
Hetian-8-MEAM1	0.02	**0.98**	Kashi-5-MED	0.08	0.06	**0.76**	0.1
Hetian-9-MEAM1	0.07	**0.93**	Kashi-6-MED	0.01	0.24	**0.74**	0.01
Hetian-11-MEAM1	0.12	**0.88**	Kashi-7-MED	0.02	0.02	**0.95**	0.01
Hetian-12-MEAM1	0.03	**0.97**	Hetian-13-MED	0.04	0.02	**0.92**	0.02
Kashi-8-MEAM1	0.14	**0.86**	Tulupan-2-MED	**0.64**	0.17	0.02	0.18
Bazhou-1-MEAM1	**0.96**	0.04	Tulupan-4-MED	**0.65**	0.12	0.14	0.1
Bazhou-3-MEAM1	**0.96**	0.04	Tulupan-5-MED	**0.8**	0.04	0.08	0.09
			Tulupan-6-MED	**0.63**	0.06	0.11	0.2
			Tulupan-7-MED	**0.89**	0.05	0.03	0.03
			Tulupan-8-MED	**0.51**	0.1	0.16	0.23
			Tulupan-9-MED	**0.45**	0.1	0.05	0.4
			Changji-MED	0.11	**0.74**	0.1	0.04
			Yining-MED	0.14	**0.75**	0.02	0.08
			Alashankou-MED	0.09	0.35	0.03	**0.54**
			Shihezi-MED	0.14	0.39	0.02	**0.45**
			Hami-1-MED	0.21	0.32	0.02	**0.46**
			Hami-2-MED	0.11	0.13	0.01	**0.74**

Bold indicate the proportion of each population assigned to the most reliable group. *K*, the number of inferred population groups; *P*, the number of populations.

#### Analysis of Molecular Variance

Basing on the subdivision by Δ*K*, AMOVA demonstrated that most of the variation came from within individuals (100.89%, 115.31%; Table 4), with *K* = 2 in MEAM1 group and *K* = 4 in MED group. Fixation indices of within populations (*F*_is_) were negative (−0.23, −0.23). It demonstrated that there was a strong disassortative mating, which was consistent with the *F*_is_ in each population (Table 5). Variation among population and groups in MEAM1 and MED population was low but significant (df = 11, *F* = 0.06, *P* = 0.000; df = 19, *F* = 0.04, *P* = 0.00; df = 1, *F* = 0.12, *P* = 0.00, df = 3, *F* = 0.03, *P* = 0.00; Table 4), which supported the groupings.

**Table 4.** Analysis of molecular variance

Source of variation	df		*F*		*P*-value		PV	
	MEAM1	MED	MEAM1	MED	MEAM1	MED	MEAM1	MED
	*K* = 2	*K* = 4	*K* = 2	*K* = 4	*K* = 2	*K* = 4	*K* = 2	*K* = 4
Among groups	1	3	0.12	0.03	0.02	0.00	12.38	3.06
Among populations	11	19	0.06	0.04	0.00	0.00	5.32	3.43
Within populations	160	434	−0.23	−0.23	1.00	1.00	−18.59	−21.79
Within individuals	173	457	−0.01	−0.15	1.00	1.00	100.89	115.31

df, degree of freedom; *K*, the number of inferred population groups based on STRUCTURE analysis; *F*, fixation index; PV, percent variation; *P*-value, probability of the significance tests.

**Table 5.** Summary data of genetic diversity among groups and populations in Xinjiang, China

Population	*N*	*n*_a_	*n*_e_	*h*	*I*	PP	PIC	*F*_is_	*P*-value
All	631	2.00 ± 0.00	1.13 ± 0.25	0.09 ± 0.13	0.15 ± 0.19	99	0.96	—	
MEAM1	174	1.77 ± 0.42	1.13 ± 0.25	0.09 ± 0.14	0.15 ± 0.20	76	0.96	—	
MED	457	1.92 ± 0.27	1.13 ± 0.25	0.09 ± 0.13	0.15 ± 0.20	91	0.96	—	
MEAM1									
Urumqi-2-MEAM1	33	1.45 ± 0.50	1.12 ± 0.24	0.08 ± 0.13	0.13 ± 0.13	45	0.95	−0.25	0.0003
Bazhou-1-MEAM1	28	1.30 ± 0.46	1.09 ± 0.23	0.06 ± 0.13	0.09 ± 0.13	30	0.92	−0.24	0.0003
Bazhou-3-MEAM1	19	1.27 ± 0.45	1.11 ± 0.25	0.07 ± 0.14	0.11 ± 0.14	27	0.93	−0.17	0.0053
Kashi-8-MEAM1	10	1.26 ± 0.44	1.10 ± 0.23	0.06 ± 0.13	0.10 ± 0.13	26	0.93	−0.29	0.0003
Hetian-13-MEAM1	21	1.38 ± 0.49	1.12 ± 0.25	0.08 ± 0.14	0.12 ± 0.14	38	0.94	−0.28	0.0003
Hetian-2-MEAM1	10	1.28 ± 0.45	1.13 ± 0.28	0.08 ± 0.15	0.12 ± 0.15	28	0.94	−0.25	0.0008
Hetian-4-MEAM1	6	1.19 ± 0.40	1.10 ± 0.24	0.06 ± 0.14	0.09 ± 0.14	19	0.93	−0.31	0.0003
Hetian-5-MEAM1	14	1.24 ± 0.43	1.09 ± 0.23	0.06 ± 0.13	0.10 ± 0.13	24	0.93	−0.34	0.0003
Hetian-6-MEAM1	5	1.21 ± 0.41	1.11 ± 0.25	0.07 ± 0.14	0.10 ± 0.14	21	0.93	−0.26	0.0047
Hetian-8-MEAM1	6	1.15 ± 0.36	1.07 ± 0.21	0.04 ± 0.12	0.07 ± 0.12	15	0.92	−0.37	0.0006
Hetian-9-MEAM1	12	1.29 ± 0.45	1.12 ± 0.25	0.07 ± 0.14	0.12 ± 0.14	29	0.94	−0.29	0.0003
Hetian-11-MEAM1	5	1.27 ± 0.44	1.14 ± 0.24	0.08 ± 0.14	0.12 ± 0.21	27	0.94	−0.13	0.0869
Hetian-12-MEAM1	5	1.22 ± 0.42	1.13 ± 0.23	0.06 ± 0.13	0.10 ± 0.20	22	0.93	−0.25	0.0025
MED									
Kelamayi-MED	40	1.48 ± 0.50	1.11 ± 0.24	0.07 ± 0.13	0.13 ± 0.13	48	0.94	−0.26	0.0003
Urumqi-2-MED	16	1.36 ± 0.48	1.11 ± 0.22	0.07 ± 0.13	0.12 ± 0.13	36	0.95	−0.26	0.0003
Changji-MED	41	1.47 ± 0.50	1.12 ± 0.25	0.08 ± 0.14	0.13 ± 0.14	47	0.95	−0.22	0.0003
Yining-MED	50	1.38 ± 0.49	1.12 ± 0.26	0.08 ± 0.15	0.12 ± 0.15	38	0.94	−0.29	0.0003
Alashankou-MED	50	1.42 ± 0.50	1.12 ± 0.25	0.08 ± 0.14	0.12 ± 0.14	42	0.94	−0.20	0.0003
Shihezi-MED	43	1.45 ± 0.50	1.13 ± 0.26	0.08 ± 0.14	0.13 ± 0.14	45	0.95	−0.23	0.0003
Hami-1-MED	16	1.31 ± 0.47	1.11 ± 0.23	0.07 ± 0.13	0.12 ± 0.20	31	0.95	−0.22	0.0003
Hami-2-MED	24	1.39 ± 0.49	1.11 ± 0.23	0.07 ± 0.13	0.12 ± 0.13	39	0.95	−0.18	0.0003
Tulupan-2-MED	10	1.25 ± 0.44	1.10 ± 0.23	0.07 ± 0.13	0.10 ± 0.13	25	0.94	−0.21	0.0025
Tulupan-4-MED	13	1.28 ± 0.45	1.10 ± 0.23	0.07 ± 0.13	0.11 ± 0.13	28	0.94	−0.21	0.0003
Tulupan-5-MED	18	1.40 ± 0.49	1.13 ± 0.25	0.08 ± 0.14	0.13 ± 0.14	39	0.95	−0.15	0.0017
Tulupan-6-MED	14	1.31 ± 0.47	1.12 ± 0.25	0.07 ± 0.14	0.12 ± 0.14	31	0.95	−0.18	0.0031
Tulupan-7-MED	18	1.35 ± 0.48	1.12 ± 0.25	0.08 ± 0.14	0.12 ± 0.14	35	0.95	−0.18	0.0003
Tulupan-8-MED	11	1.24 ± 0.43	1.10 ± 0.24	0.06 ± 0.14	0.10 ± 0.14	24	0.94	−0.19	0.0097
Tulupan-9-MED	8	1.29 ± 0.45	1.13 ± 0.26	0.08 ± 0.15	0.13 ± 0.15	29	0.95	−0.15	0.0361
Kashi-2-MED	11	1.35 ± 0.48	1.12 ± 0.24	0.08 ± 0.14	0.13 ± 0.14	35	0.95	−0.19	0.0008
Kashi-1-MED	33	1.47 ± 0.50	1.12 ± 0.23	0.08 ± 0.14	0.13 ± 0.14	47	0.95	−0.24	0.0003
Kashi-3-MED	11	1.33 ± 0.47	1.11 ± 0.22	0.07 ± 0.13	0.12 ± 0.13	33	0.94	−0.25	0.0003
Kashi-4-MED	6	1.28 ± 0.45	1.12 ± 0.24	0.08 ± 0.14	0.12 ± 0.14	28	0.95	−0.08	0.2736
Kashi-5-MED	9	1.28 ± 0.45	1.09 ± 0.20	0.06 ± 0.12	0.11 ± 0.12	28	0.94	−0.16	0.0219
Kashi-6-MED	4	1.21 ± 0.41	1.12 ± 0.26	0.07 ± 0.15	0.11 ± 0.15	21	0.94	−0.19	0.1000
Kashi-7-MED	5	1.18 ± 0.39	1.10 ± 0.24	0.06 ± 0.14	0.09 ± 0.14	18	0.93	−0.31	0.0119
Hetian-13-MED	6	1.18 ± 0.39	1.10 ± 0.24	0.06 ± 0.14	0.09 ± 0.14	45	0.95	−0.18	0.0433

*N*, sample size; *n*_a_, observed number of alleles; *n*_e_, effective number of alleles (Kimura and Crow 1964); *h*, Nei’s (1973) gene diversity; *I*, Shannon’s Information index (Lewontin 1972). *F*_is_, inbreeding coefficient. PP, polymorphism position; PIC, polymorphism information content.

#### Mantel *t*-Test

Mantel test analysis of the matrix (Ln Km) based on Δ*K* subdivision showed that there was no correlation between geographical and genetic distances among *B. tabaci* complex (*R* = 0.42, *P* = 1.00), or in MED (*R* = 0.49, *P* = 1.00) and MEAM1 (*R* = 0.44, *P* = 1.00) group or subgroup (Table 6). MEAM1-group2 and MED-group3’s data were not available as there were only two populations in these groups.

**Table 6.** The correlations between genetic distance and geographic distance among *Bemisia tabaci* populations in Xinjiang, China

	All	MEAM1 group			MED group				
		*K* = 2			*K* = 4				
		All	Subgroup 1	Subgroup 2	All	Subgroup 1	Subgroup 2	Subgroup 3	Subgroup 4
*R*	0.42	0.44	0.42	Na^*a*^	0.49	0.20	−0.22	Na	0.05
*P*	1.00	1.00	1.88	Na	1.00	0.78	−1.22	Na	0.50
*T*	9.82	3.10	0.97	Na	5.60	0.78	0.11	Na	0.69

*R*, matrix correlation, *P*, prob. random *Z* < obs. *Z*; *T*, approximate Mantel *t*-test.

^*a*^Na, not available because there was only two populations in this group.

#### Neighbor-Joining Tree

**Fig. 3.**
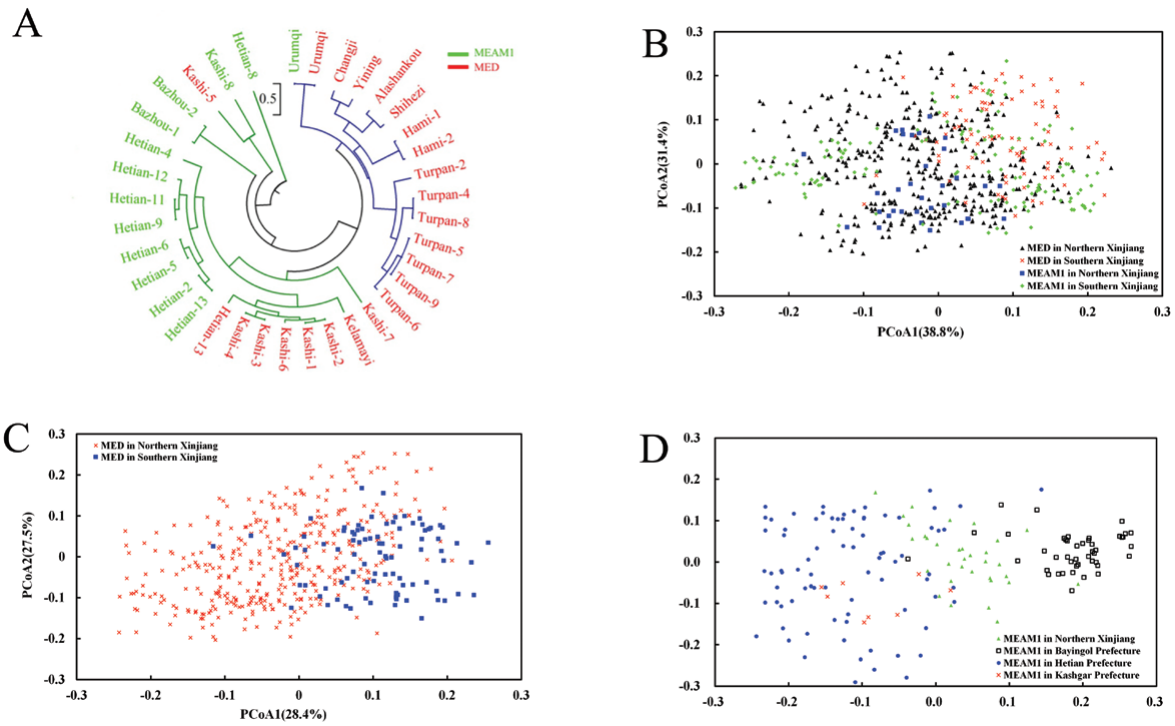
Genetic relationship analyzed by a unrooted neighbor-joining (NJ) phylogenetic tree (A) and principal coordinate analysis (PCoA) plot of *Bemisia tabaci* complex (B), MED (C), and MEAM1 (D) population in Xinjiang. For NJ trees, green and blue branches indicate population from Southern and Northern Xinjiang, respectively.

The neighbor-joining tree (Fig. 3A) showed that most of the MED and MEAM1 population were clustered separately, except for the Kashi, Kelamayi, and Urumqi population. The Kashi-5-MED and Urumqi-MEAM1 population were clustered with Kashi-8-MEAM1 and Urumqi-MED. The rest of the Kashi-MED population (Kashi-1, Kashi-2, Kashi-3, Kashi-4, Kashi-6, Kashi-7, and Kashi-13), Kelamayi-MED in northern-Xinjiang, and Hetian-MEAM1 in Southern Xinjiang converged into one branch. Nei’s genetic clustering results showed that there was no significant correlation between genetic relationship and the regional distribution of *B. tabaci* in Xinjiang.

#### Principal Coordinate Analysis

The phylogenetic tree has shown that most of MED and MEAM1 populations could be clearly separated according to geographic clustering. Thus, we verified the relationships between MED and MEAM1 samples with a PCoA plot. Based on 99 alleles of five SSR primers, the top three principle components accounted for 55.9, 67.9, and 70.2% of the SSR variation in MED, MEAM1, MED, and MEAM1 complex. There were no clear boundaries between MED and MEAM1 samples. However, the MED samples from Southern Xinjiang were mainly located at the right side of the coordinate axis, whereas the MEAM1 samples from Northern Xinjiang were mainly located at the bottom (Fig. 2B). We further tested whether MED and MEAM1 samples could be separated by geographic isolation. Results show that MED samples that grouped by sampling sites were highly highly dispersed but can be partially separated into Northern and Southern Xinjiang, with the large Tianshan Mountain potentially serving as a geographical barrier for the two groups (Fig. 2C). MEAM1 samples were clearly separated, especially those from Southern Xinjiang. Interestingly, MEAM1 in Northern Xinjiang occupied the center which separated the samples of Bayingol from those of Hotan and Kashgar.

MEAM1 *B. tabaci* in northern Xinjiang showed clear separation from those in Hotan, Bazhou, and Kashgar area. MED *B. tabaci* from northern Xinjiang was intersected by those from Bazhou and Hotan areas. The results showed further that MED *B. tabaci* individuals and MED *B. tabaci* from southern Xinjiang were clearly clustered, whereas those from northern Xinjiang were more dispersed. The range of MED from northern Xinjiang overlaps with that of MED *B. tabaci* from southern Xinjiang.

#### Gene Flow

**Fig. 4.**
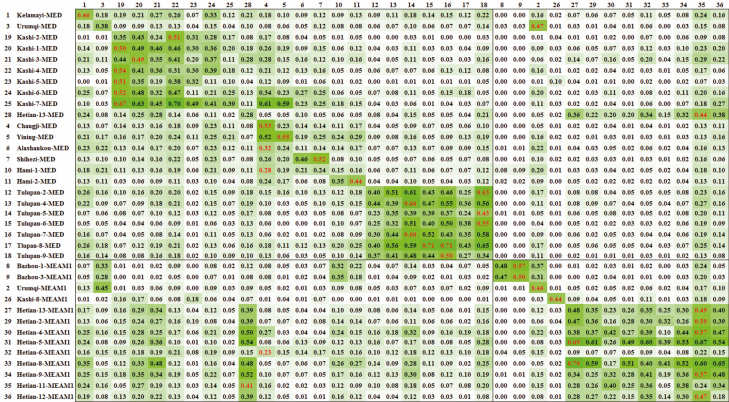
Means of individual assignment probabilities from (rows) and into (columns) each population of *Bemisia tabaci* in Xinjiang. Populations with immigrating individuals are given in rows; populations with emigrating individuals in columns. Note: Highest assignment probability in rows and means of migration probabilities in each column > 0.20 were red. The green background color deepened as the probability got higher. Subdivision of MED and MEAM1 groups were framed, respectively.

The GENECLASS 2.0 assigned individual probabilities to evaluate the degree of migration and the migration direction of MED or MEAM1 groups based on Δ*K* (Fig. 4). The results indicate that individuals migrated between MED and MEAM1 *B. tabaci* populations. Most of the highest probabilities were self-assigned in each subdivision or even in local areas. However, probabilities of migration from Urumqi-MEAM1 to Urumqi-MED (0.47), Hetian-11-MEAM1 to Hetian-13-MED (0.44), Changji-MED to Hetian-6-MEAM1 (0.23), Changji-MED to Alashankou-MED (0.32), Changji-MED to Hami-MED (0.28), and Hetian-13-MED to Hetian-11-MEAM1 (0.41) were higher than self-assigned probabilities in their subdivisions.

**Fig. 5.**
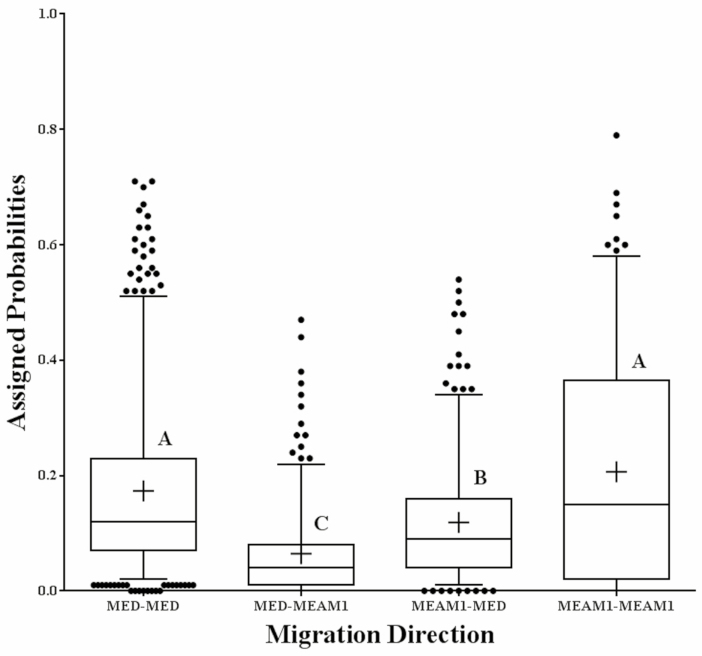
Box diagram of assigned probabilities of migration from MED to MED, MED to MEAM1, MEAM1 to MED, and MEAM to MEAM1. Probabilities above 95% and below 5% were depicted as dots and 5% and 95% threshold as bars. Horizontal lines and in box indicate quantiles and medians. ‘+’ expresses means. Capital letters mean significant levels at *P* < 0.001.

To predict the immigrant origins, we calculated the means of migration probabilities in each column (Fig. 4). Interestingly, we found that populations of Kashi-3-MED (0.22), Kashi-4-MED (0.22), Kashi-5-MED (0.21), Kashi-3-MED (0.20), Hetian-13-MED (0.21), Turpan-5-MED (0.21), Hetian-11-MEAM1 (0.26), and Hetian-11-MEAM1 (0.20) had a relatively high value. Migration assigned probabilities from MED to MED, MED to MEAM1, MEAM1 to MED, and MEAM1 to MEAM1 are also depicted as box diagrams (Fig. 5). Results showed that there was no difference between migration assigned probabilities from MED to MED (mean ± SE = 0.174 ± 0.007) and MEAM1 to MEAM1 (0.206 ± 0.015; *F* = 0.007, *P* = 0.977), but there were significant differences between MED to MED and MED to MEAM1 (0.065 ± 0.005; *F* = 0.184, *P* < 0.001), MED to MED and MEAM1 to MED (0.119 ± 0.006; *F* = 0.077, *P* < 0.001), MEAM1 to MEAM1 and MED to MEAM1 (*F* = 0.1844, *P* < 0.001), and finally, MEAM1 to MEAM1 and MEAM1 to MED (*F* = 0.083, *P* < 0.001).

To further analyze the gene flow between MED and MEAM1 populations in local regions, Kashi and Hetian populations were selected since they were coexisting populations and there were plenty of sampling points. Kashi-8-MEAM1 was the only MEAM1 population chosen for the SSR analysis out of six randomly selected Kashi populations. Hetian-13-MED was the only population chosen out of 10 randomly selected Hetian populations. MED and MEAM1 showed different migration patterns. For Kashi-8-MEAM1 population, the assigned probability was self-assigned (0.44) and the rest of the Kashi-MED populations were mainly migrants from Kashi-2-MED (0.35–0.67). As for Hetian-13-MED, the gene flows from Hetian-11-MEAM1 (0.44), Hetian-12-MEAM1 (0.38), Hetian-13-MEAM1 (0.36), Hetian-6-MEAM1 (0.34) were higher than the self-assigned (0.28). The gene flow from Hetian-13-MED population to other populations was high (0.39–0.52), except for Hetian-6-MEAM1 population, which received migrants mainly from Changji-MED. Apart from Hetian-6-MEAM1 and Hetian-11-MEAM1, the Hetian-MEAM1 populations received gene flows mainly from Hetian-11-MEAM1 (0.47–0.67) and Hetian-13-MEAM1 (0.69–0.79).

## Discussion

MED *B. tabaci* has spread and gradually replaced the ecological niche of MEAM1 as a dominant cryptic species in many provinces since it was first found in Yunnan in 2004 (Chu et al. 2005). After 22 yr of *B. tabaci* invasion in Xinjiang, CAPs were used to identify MED and MEAM1 cryptic species in this study, and the randomly chosen SSR samples were also used for sequencing COI gene (data not shown) to verify CAP-based identification. Based on the results of CAPs, we found that MED *B. tabaci* was mainly distributed in northern Xinjiang and was in agreement with Cao et al. (2013). We also found that in southern Xinjiang, a higher proportion of MEAM1 existed, there was a wide percentage difference between northern (MED: 84%) and southern (MED: 28%) Xinjiang. The investigation revealed that the most serious damage occurred in Turpan area and the MED was the dominant crop pest. This was attributed to local climatic conditions, planting patterns, and field management. The samples from Shihezi, Alashankou, Changji, and Aler were collected in flowers and were identified and confirmed as MED-byrelated studies. The spread of MED *B. tabaci* is related to the introduction of flowers (Chu et al. 2005). MEAM1 was the main cryptic species in southern Xinjiang including western part of Bazhou, parts of Hotan and Kashgar. This phenomenon could be attributed to the adjacent planting of local vegetable greenhouses, cotton fields, and with frequent flower transport and trade.

We were interested in the invasion pathways of MEAM1 and MED *B. tabaci* and the effect of substitutions on the internal genetic diversity of *B. tabaci*. We used five microsatellite primers to analyze the genetic relationship between MED and MEAM1 populations. There was low genetic diversity in *B. tabaci* in Xinjiang and low genetic variation between populations. The main sources of genetic variation between MED and MEAM1 *B. tabaci* were interspecific genetic differences and geographical isolation. There was no correlation between the geographical and genetic distances in *B. tabaci*. The reason is that the man-made transmission factor of *B. tabaci* is larger than that of the natural transmission. To block ongoing dispersal, strict detection and flower quarantine regulations need to be enforced. Based on the AMOVA and the *F*_ST_ value, *B. tabaci* populations in Xinjiang had highly significant differentiation. The correlation between the geographical distance (LN Km) and the genetic distance of the whitefly populations was analyzed using Mantel test. Mantel’s *t*-test demonstrated that there was no relationship between geographical distance and genetic distance (Table 6). Δ*K* statistic divides MEAM1 into two subgroups and into four subgroups for MED. As the provincial capital, Urumqi may be the main source of MED whitefly transmission, while MEAM1 invasion pathways may be more varied. NJ-tree and PCoA could neither distinguish MED and MEAM1 nor separate the northern and southern Xinjiang into two clusters well (Fig. 3). This may be explained by the gene flow between a few MED and MEAM1 populations, such as Hetian-13-MED, Urumqi-2-MEAM1, and Changji-MED (Fig. 4). The unique characteristics of irrigation agriculture and oasis agriculture in Xinjiang, at the same time, the large desert areas surrounding cities, provide geographical barrier for whitefly transmission. Thus, human-based transmission becomes the main mode of transmission for *B. tabaci*. MED populations had a stronger gene flow toward MEAM1 populations. The probabilities in MEAM1 were mainly self-assigned, indicating that the MEAM1 had a reproductive disadvantage to MED. This can result from pesticide resistance (Roditakis et al. 2009, Wu et al. 2010) or asymmetric mating interaction (Liu et al. 2007).
